# The impact of residential status on cognitive decline among older adults in China: Results from a longitudinal study

**DOI:** 10.1186/s12877-017-0501-9

**Published:** 2017-05-15

**Authors:** Hanzhang Xu, Matthew E. Dupre, Danan Gu, Bei Wu

**Affiliations:** 1 Duke University School of Nursing, Durham, NC USA; 2 Duke Global Health Institute, Duke University Medical Center, Durham, NC USA; 3 Department of Community and Family Medicine, Duke University Medical Center, Durham, NC USA; 4 Department of Sociology, Duke University, Durham, NC USA; 5 Duke Clinical Research Institute, Duke University Medical Center, Durham, NC USA; 6 United Nations Population Division, New York, NY USA; 7 New York University Rory Meyers College of Nursing, New York, NY USA

**Keywords:** China, Cognitive decline, Older adults, Residential status

## Abstract

**Background:**

Residential status has been linked to numerous determinants of health and well-being. However, the influence of residential status on cognitive decline remains unclear. The purpose of this research was to assess the changes of cognitive function among older adults with different residential status (urban residents, rural-to-urban residents, rural residents, and urban-to-rural residents), over a 12-year period.

**Methods:**

We used five waves of data (2002, 2005, 2008/2009, 2011/2012, and 2014) from the Chinese Longitudinal Healthy Longevity Survey with 17,333 older adults age 65 and over who were interviewed up to five times. Cognitive function was measured by the Mini Mental State Examination (MMSE). Multilevel models were used regarding the effects of residential status after adjusting for demographic characteristics, socioeconomic factors, family support, health behaviors, and health status.

**Results:**

After controlling for covariates, significant differences in cognitive function were found across the four groups: rural-to-urban and rural residents had a higher level of cognition than urban residents at baseline. On average, cognitive function decreased over the course of the study period. Rural-to-urban and rural residents demonstrated a faster decline in cognitive function than urban residents.

**Conclusions:**

This study suggests that residential status has an impact on the rate of changes in cognition among older adults in China. Results from this study provide directions for future research that addresses health disparities, particularly in countries that are undergoing significant socioeconomic transitions.

**Electronic supplementary material:**

The online version of this article (doi:10.1186/s12877-017-0501-9) contains supplementary material, which is available to authorized users.

## Background

The aging population in China has grown dramatically over the past several decades. Between 2000 and 2013, the number of Chinese aged 65 and older increased from 90 million to more than 200 million [1]. By 2050, this segment of the population is projected to increase to more than 300 million and will account for approximately 30% of the entire population [1]. As the number of older adults increases, so has the number of elderly living with some form of cognitive impairment. For example, a recent epidemiological study showed that the prevalence of mild cognitive impairment (MCI) in China was 20.1% among older adults aged 60 or above [2]. In addition, it is estimated that around 6% of the older adults with MCI progress to dementia annually [3]. Such increase in cognitive impairment in aging populations have major societal and human implications [4]. Studies show that persons with cognitive impairment are at greater risk of physical disabilities, disease comorbidities, hospital admissions, and subsequent mortality than their cognitively normal counterparts [4–7]. Therefore, recent research has increasingly sought to understand the factors contributing to cognitive decline in older adults.

In the context of an aging population, China has also undergone a rapid urbanization due to its significant economic growth and social mobility in recent decades [8]. The share number of urban population increased from 19.4% in 1980 to 54.4% in 2014 [9]. One of the key contributors to the growing urban population is internal (rural-to-urban) migration among adult populations [8]. In 2010, the number of internal migrants in China reached 221 million—nearly 1/6 of the nation’s total population—and is projected to double in the next 10 years [10]. The implications of these massive demographic changes are not without health consequences. Indeed, there is now increasing evidence to suggest significant health disparities among urban residents, rural residents, and internal migrants in China [11, 12]. Although findings have been mixed, most studies have demonstrated a “healthy migrant effect”—the observed finding that people who migrate are often healthier than those who did not migrate [13, 14]. Accordingly, research has shown that rural-to-urban migrants in China report better self-rated health than their rural counterparts [13]. Those who migrate to urban areas also exhibit lower rates of acute illnesses and disabilities relative to native urban residents [15, 16]. However, almost all these previous studies used cross-section data, research is lacking in our understanding of changes in health status, such as cognitive function, in relation to changes of residential status, as the results of urbanization and migration in China. Furthermore, we know surprisingly little about the potential factors associated with stability (or change) in urban-rural residence that may influence trajectories of cognitive function in later life.

This study uses multiple waves of the Chinese Longitudinal Healthy Longevity Survey (CLHLS) to examine age-related trajectories of cognitive function among older adults living in urban and rural areas of China. We investigate whether and to what extent stability or change in residence—i.e., native urban residence, native rural residence, rural-to-urban residence, and urban-to-rural residence—is associated with changes in cognitive function over a period of a decade. We also examine a wide breadth of socioeconomic, family support, behavioral, and health-related factors that may be contributing to the associations. The public health and policy implications of the findings are discussed.

Since the economic reforms in 1979, China has experienced significant economic growth and social changes [17]. During this period, an acceleration of urbanization occurred throughout eastern regions of the nation and the number of rural-to-urban migrants increased steadily through the beginning of the twenty-first century [18, 19]. The demographic shift occurring from widespread geographic mobility in China has been described as one of most extensive migrations in the human history [20]. Furthermore, many of those residing in China also experienced varying degrees of hardship from World War II, the Cultural Revolution, periods of hunger or starvation, limited educational opportunities, and other consequences of dynamic changes in socio-political institutions [21]. Although the patterns and processes of migration have been well documented in the complex context of China, its association with cognitive function in later life is understudied.

### Migration and cognitive function

A significant number of studies have focused on the relationship between international migration (i.e., immigration) and cognitive function [22–25]. However, the results from this literature are often inconsistent and currently inconclusive—with some studies indicating that there is no association between migration and cognitive status [24, 26, 27]. Far fewer studies have focused exclusively on migration within a country (internal migration) and the evidence is similarly contradictory. From the limited research that exists, there is some support for the healthy migrant hypothesis [13, 28]—which argues that migrants are typically healthier than those living in the receiving location (often the attributed to the selection of healthy migrants). For example, research from India showed that adults who migrated from rural to urban areas had lower rates of dementia than adults who resided in urban areas for long periods of time [28]. However, this finding was not supported by another study that demonstrated no difference in cognitive function between migrants and non-migrants [29]. Nevertheless, both studies were limited by cross-sectional designs and only studied participants from a small geographic area.

To date, no existing studies have examined a longitudinal association between internal migration and changes in cognitive function among older adults in China. We argue that migration is an understudied social determinant of health that operates through complex and multifactorial pathways over time. From a life course perspective, migration (or the change of residential status) is associated with a number of socioeconomic, occupation, environment, social support, behavioral, and health-related factors that can have a cumulative and lasting impact on later-life trajectories of cognitive health.

An individual’s socioeconomic status (SES) might change along with the migration process. For example, previous studies showed that in developing countries, a large amount of rural residents migrated to urban settings for better education and working opportunities [10, 30]. These advantages in SES such as education and income are considered to be protective factors of cognitive decline [31–34].

However, there may be some disadvantages related to migration. For example, changes in an individual’s health behaviors are often observed from a migrant population. Rural-to-urban migrants were more likely to adapt to westernized life styles: high calorie intake, physical inactivity, and sedentary employment [35, 36]. These unhealthy life styles are considered as risk factors of cognitive impairment [37–39]. In addition, these factors not only directly affect an individual’s cognitive function but also increase the likelihood of developing chronic diseases such as hypertension and diabetes that have been shown to produce negative effects on later life cognition [40, 41].

Previous research reported that migrants often experienced various stressful life events, such as separating from their families, during and after the migration process [42]. In addition, a number of studies well documented the hostility and discrimination that migrants received. For example, rural-to-urban migrants are often denied access to many of the social welfare programs such as health insurance and unemployment benefits that are available to their urban counterparts, even if they are doing the same job [10]. The perceived discrimination and family separation may result in lack of social support, which has been found to have negative impact on an individual’s cognitive function [43, 44].

The change in residential status may also result in changes in living and working environments. Literature suggested that people work in agricultural settings are more likely to expose pesticide that was related to decline of cognitive function [45]. In addition, open fire cooking, a major source of indoor pollution, is still more common among rural households especially in developing countries [46–48]. Indoor pollution has been shown to be associated with poorer cognitive function [49]. Therefore, moving from a rural to an urban setting are likely to have improved living and working environments, which may yield a positive influence on cognitive function.

Besides all the factors that might change during the change of residential status, other factors also determine an individual’s cognitive function. For example, a growing body of literature has demonstrated the association between physical function and cognitive function [50, 51]. Age is also a strong risk factor associated with cognitive decline [52]. Compared with male, female population reported a worse cognitive function especially in the oldest old age range [53, 54].

Overall, individual’s SES, psychological well-being, health status, and health behaviors influence cognitive function both directly and indirectly. The migration process might change some of these factors therefore influence an individual’s cognitive function.

### Hypotheses

To our knowledge, this is the first study that examines how migration contributes to the cognitive function trajectories over a specific period of time among the CLHLS study participants. We expect that migration will play an important role in the differences in cognitive function trajectories among Chinese older adults. Based on prior empirical evidence and a life course perspective we posited the following hypotheses for analysis: Hypothesis 1: Native rural residents will tend to exhibit worse cognitive function and a faster rate of cognitive decline than native urban residents. Hypothesis 2: Compared to urban residents, both rural-to-urban residents and urban-to-rural residents will show similar cognitive function at baseline but a faster rate of cognitive decline than native urban residents.

## Methods

### Data

We used multiple waves of data from the Chinese Longitudinal Healthy Longevity Survey (CLHLS) for this study. The CLHLS was designed to collect information on factors related to health and longevity in older adults from 22 provinces in mainland China [55]. The survey was administered every two to three years between 1998 and 2014. Beginning in 2000, newly recruited samples were added to replenish and augment the sample of oldest-old adults; and a smaller comparative sample of adults aged 65–80 years was added starting in 2002. The details of the CLHLS sampling design, response rates, attrition, and data quality have been described extensively elsewhere [55].

**Fig. 1:**
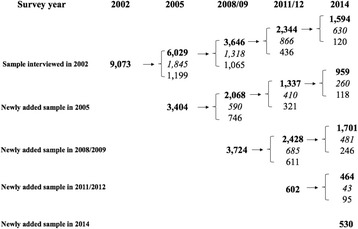
Structure of the study sample. Note: Numbers in bold font indicate survivors at each wave. Numbers in italic font indicate deceased persons. Numbers in normal font indicate respondents who were lost to follow-up

The current study draws from data collected in 2002, 2005, 2008–2009, 2011–2012, and 2014. Participants who were 90 years or older at baseline (*n* = 17,304) were excluded to reduce possible selection bias and an additional 12 participants were excluded because of missing data on cognitive function. Therefore, the total analytic sample consisted of 17,333 participants aged 65–90 at baseline who contributed 39,900 observations over the study period. Figure 1 illustrates the structure of the analytic sample by survey year, initial interview year, and survival status across waves.

### Measures

#### Cognitive function

The Chinese version of the Mini-Mental State Examination (MMSE) was used to measure cognitive function [56, 57]. The MMSE captured four dimensions of older adults’ cognitive ability: cognitive orientation, calculation, recall, and language. As previously documented, several items in the Chinese version of MMSE were modified to improve their meaning and cultural acceptability [58]. The reliability and validity of the MMSE in the CLHLS has been established in prior study [58]. In particular, previous research showed that participants were more likely to be unable to answer relatively difficult tasks when they exhibited poor health and/or existing cognitive limitations [57]. Therefore, following prior research, we categorized responses of “unable to answer” as incorrect answers [33, 59, 60]. This approach is widely used in previous studies and will not introduce potential bias [33, 60]. We also explored alternative approaches to handle the cases for “unable to answer,” such as adding an additional variable in the analyses to indicate whether the participant was unable to answer any question. We found that this approach yielded very similar results as the approach used in this study. A total MMSE score was calculated and ranged from 0 to 30, with lower scores indicating poor cognitive ability.

#### Residential status

Residential status was ascertained by asking participants about their residential location in early life and in adult life at each wave. Categorical variables were used to indicate participants who were: 1) rural, 2) urban, 3) rural-to-urban, and 4) urban-to-rural. Persons born in rural areas and are currently living in rural areas are considered “rural;” and persons born in urban areas and are currently living in urban areas are considered “urban.” Participants who were born in rural areas and currently living in urban areas were defined as “rural-to-urban” residents. Finally, participants who were born in urban areas and currently living in rural areas were defined as “urban-to-rural” residents. The categorical variables are time-varying in the prospective analyses and account for changes in adult residential location during the study period.

#### Covariates

Based on existing literature, we included numerous covariates that are associated with residential status and/or cognitive function (see Table 1 for details). Demographic characteristics  included age (in years), gender (male = 1), and ethnicity (Han = 1). Socioeconomic factors included childhood SES, years of education (no education, 1–6, or 7+ years), primary lifetime occupation (professional/administrative = 1), and whether the respondent is economically independent (yes = 1). Family support factors included the participants’ marital status (married = 1) and proximity to their children (co-residence with children or having a child living in the same village/neighborhood [high proximity] =1). Behavioral factors included smoking (current or ever smoked = 1), regular consumption of vegetables (yes = 1) and fish (yes = 1), and routine exercise (yes = 1). Finally, covariates for health status included having any disability in activities of daily living (ADLs) and instrumental activities of daily living (IADLs), and having any diagnosed chronic disease.

**Table 1:** Definition of Covariates

Variable name	Data collection time points	Definitions
Demographic characteristics		
Age	2002–2014	Continuous variable
Gender	2002	Male = 1, female = 0
Ethnicity	2002	Han = 1, non-Han minorities = 0
Socioeconomic Factors		
Education	2002	No education = 0, received 1–6 years schooling = 1, received 7 years or more education = 2
Prof./admin. Occupation	2002	Professional work = 1, others = 0
Economic independence	2002	A dichotomized variable with 1 if the respondent’s primary financial source was from own work or pension, 0 if not
Childhood SES	2002	a continuous variable ranging from 0 to 4 with 1 point each if the respondent was obtained \adequate medical services, or went to bed without hunger, or if both parents were alive at age 10, or the father’s occupation was white collar
Family Support Factors		
Marital status	2002–2014	Married = 1, others = 0
High proximity to children	2002–2014	A dichotomized variable with 1 if the respondent was co-residing with children or had at least 1 biological children living in the same village or street block
Behavioral Factors		
Current smoking	2002–2014	Yes = 1, no = 0
Exercises regularly	2002–2014	Yes = 1, no = 0
Consuming vegetables	2002–2014	A dichotomized variable with 1 if the respondents said that they consumed vegetables “almost every day”, 0 if answered “occasionally” or “rarely or never.”
Consuming fish	2002–2014	A dichotomized variable with 1 if the respondents said that they consumed fish “almost every day”, 0 if answered “occasionally” or “rarely or never.”
Health Status		
Any chronic condition	2002–2014	A dichotomized variable with 1 if the respondents said that they reported hypertension, a pulmonary disorder, heart attack, or cerebrovascular disease.
Any ADL disability	2002–2014	A dichotomized variable with 1 if the respondents said that they needed any help in performing the following tasks: bathing, dressing, toileting, indoor transferring, maintaining continence, and eating.
Any IADL disability	2002–2014	A dichotomized variable with 1 if the respondents said that they needed any help in performing the following tasks: visiting neighbors, washing clothes, walking one kilometer, shopping, cooking, lifting 5 kg, crouching and standing up three times, and using public transportation
Attrition	2002–2014	Survivor = 0, loss to follow-up = 1, deceased = 2

Note: all covariates are time varying with the exception of participants’ demographic and socioeconomic factors.

Missing data on study measures was minimal in the CLHLS sample (less than 1%). Various strategies were assessed to address missing data (e.g., imputation, mean assignment) and the results were very similar. Therefore, the current analysis uses listwise deletion to handle missing data.

### Analyses

Baseline characteristics of study participants were examined across the four residence groups. Group differences were tested using one-way ANOVA for continuous variables and chi-square tests for categorical variables. To examine changes in older adults’ cognitive function across age, we used two-level multilevel models with maximum likelihood estimation—using an *xtmixed* procedure in Stata for analyzing longitudinal data with attrition. First, we fit unconditional models with fixed and random linear (age) and quadratic (age2) functions that were added to the intercept-only model. Tests of model fit in preliminary analyses based on BIC values (see Additional file 1: Table S1) indicated that a linear function best parameterized the pattern of cognitive decline in the data. At Level 1, we estimated a linear trajectory of individual-level changes in cognitive function as a function of increasing age. We estimated between-individual effects in the age trajectories of cognitive function at Level 2. All models also controlled for mortality and loss to follow-up. The multivariate analyses were conducted in several steps. First, demographic characteristics were included to adjust for the participants’ background characteristics. Next, we included a series of models to assess how socioeconomic resources, family support, health behaviors, and health-related factors contributed to the associations. A final model was tested that included all study covariates. All of the covariates were included in the model at either level 1 (time-varying) or level 2 (time-invariant). We also estimated goodness of fit indices (i.e., deviance statistic, Akaike Information Criterion [AIC], and Bayesian Information Criterion [BIC]) to compare fit across the non-nested models [61].

Sampling weights were not used in the analyses because previous research indicates that the CLHLS sampling strategy was not designed as a nationally representative sample [55]. In addition, all multivariate analyses included variables used in the sampling weights (e.g., age, sex, and urban-rural residence) to produce unbiased estimates and avoid inflated standard errors [62]. Stata version 14.2 was used for all analyses.

## Results

### Descriptive statistics

Baseline sample distributions are reported in Table 2 for the overall sample and by residential status. Overall, the average age of participants was approximately 78 years, the small majority were men (51%), and most were non-Han ethnicity (94%). More than half of the entire sample was rural residents (55.25%) and most of the respondents (85.3%) did not migrate during the study period. Compared with other residential groups, rural residents had significantly lower levels of education, were less likely to work in professional/administrative occupations, and were less financially independent. Rural residents were also less likely to consume vegetables and fish, less likely to engage in regular exercise, and more likely to smoke. Urban residents—and rural-to-urban residents—were younger, more likely to work in professional/administrative jobs, and were more independent financially. However, rural-to-urban residents showed the worst childhood SES among the 4 groups. Urban residents also reported the highest rates of diagnosed chronic disease and ADL disability; whereas urban-to-rural residents reported somewhat higher rates of having at least one IADL disability. Overall, the mean score of MMSE was 25.7 at baseline, with rural residents exhibited the lowest MMSE scores, followed by rural-to-urban residents, and urban residents exhibited the highest scores.

**Table 2:** Baseline Descriptive Characteristics of the CLHLS Study Sample

		Residence				
Variables	Total (*n* = 17,333)	Urban (*n* = 2325)	Rural to Urban (*n* = 5059)	Rural (*n* = 9578)	Urban to Rural (*n* = 371)	*P* value
Demographic characteristics						
Age*, mean* (SD)	77.9 (7.87)	77.3 (8.10)	78.3 (7.80)	77.5 (7.94)	77.8 (7.84)	<0.001
Male,%	51.40	51.74	49.99	82.05	52.02	0.122
Han ethnicity,%	93.69	96.00	95.77	91.98	94.88	<0.001
Socioeconomic Factors						
Education,%						
No education	52.28	31.14	49.38	59.11	47.98	<0.001
6 years of education	34.41	35.27	35.93	33.44	33.15	
7+ years of education	13.31	33.59	14.69	7.44	18.87	
Prof./admin. Occupation,%	10.62	25.55	15.81	4.20	11.86	<0.001
Economic independence,%	39.96	68.00	48.73	28.43	42.32	<0.001
Childhood SES*, mean (SD)*	1.73 (0.77)	1.80 (0.85)	1.72 (0.78)	1.73 (0.74)	1.86 (0.76)	<0.001
Family Support Factors						
Married,%	50.22	53.68	49.26	49.79	52.56	0.002
High proximity to children,%	83.29	70.67	77.98	89.13	83.83	<0.001
Behavioral Factors						
Current smoking,%	24.09	21.29	21.82	26.02	22.64	<0.001
Exercises regularly,%	34.86	55.61	46.67	23.07	48.25	<0.001
Consumes vegetables,%	89.35	91.57	90.16	88.36	90.16	<0.001
Consumes fish,%	30.65	41.20	35.11	25.42	38.54	<0.001
Health Status						
Any chronic condition,%	60.53	69.25	64.18	56.59	57.95	<0.001
Any ADL disability,%	12.10	15.14	15.04	9.95	8.63	<0.001
Any IADL disability,%	49.91	46.62	50.56	50.29	51.75	0.007
MMSE, *mean* (SD)	25.7 (5.93)	26.85 (5.09)	25.87 (5.90)	25.26 (6.10)	26.30 (5.68)	<0.001
Died during study period,%	41.12	32.56	44.57	41.12	47.71	<0.001 <0.001
Loss to follow-up,%	28.60	45.42	35.86	19.25	33.42	

*Abbreviations*: *SD* standard deviation

### Multilevel models of change in cognitive decline

Table 3 presents the results from the multilevel models estimating the associations between residential status and changes in cognitive function. Model 1 adjusted for demographic characteristics and showed that rural residents had significantly lower MMSE scores (coefficient = −0.78; *p* < .001) than urban residents. In addition, rural residents and rural-to-urban migrants had significantly faster declines in cognitive function than urban residents. These patterns were largely unchanged after adding participants’ family support (Model 3) and health status (Model 5) into the models.

**Table 3:** Estimates of Coefficients for Residential Status in Mixed Effect Model on Cognitive Performance, CLHLS 2002–2014

	Model 1	Model 2	Model 3	Model 4	Model 5	Model 6
Age^a^	−0.21 (0.01)***	−0.18 (0.02)***	−0.20 (0.01)***	−0.18 (0.01)***	−0.11 (0.01)***	−0.07 (0.01)***
Residential status (Ref: urban residents)						
Rural-to-urban residents	0.06 (0.17)	0.31 (0.17)	−0.05 (0.17)	0.26 (0.17)	−0.01 (0.17)	0.50 (0.17)**
Rural residents	−0.78 (0.16)***	0.38 (0.17) *	−0.75 (0.16)***	0.08 (0.16)	−0.68 (0.16)***	0.42 (0.16)**
Urban-to-rural residents	−0.47 (0.38)	−0.04 (0.38)	−0.45 (0.38)	−0.09 (0.38)	−0.15 (0.37)	0.31 (0.36)
Age * Residential status (Ref: Age *urban residents)						
Age *Rural-to-urban residents	−0.06 (0.01)***	−0.06 (0.01)***	−0.06 (0.01)***	−0.06 (0.01)***	−0.06 (0.01)***	−0.07 (0.01)***
Age *Rural residents	−0.03 (0.01)**	−0.04 (0.01)**	−0.03 (0.01)**	−0.06 (0.01)***	−0.05 (0.01)***	−0.07 (0.01)***
Age *Urban-to-rural residents	−0.01 (0.03)	−0.01 (0.03)	−0.01 (0.03)	−0.03 (0.03)	−0.05 (0.02)	−0.05 (0.02)
Demographic characteristics						
Male	1.25 (0.06)***	0.58 (0.07)***	1.12 (0.06)***	1.03 (0.07)***	0.91 (0.06)***	0.28 (0.07)**
Han ethnicity	−0.18 (0.13)	−0.22 (0.13)	−0.18 (0.13)	−0.28 (0.13)*	0.07 (0.12)	−0.01 (0.12)
Socioeconomic Factors						
Education (Ref: no education)						
1–6 years of education		1.12 (0.07)***				0.94 (0.07)***
7+ years of education		1.49 (0.11)***				1.23 (0.11)***
Prof./admin. Occupation		0.22 (0.11)*				0.11 (0.10)
Economic independence		0.66 (0.07)***				0.35 (0.06)***
Childhood SES		0.20 (0.04)***				0.16 (0.04)***
Family Support Factors						
Married			0.50 (0.07)***			0.25 (0.06)***
High proximity to offspring			−0.09 (0.07)			0.10 (0.07)
Behavioral Factors						
Current smoking				0.30 (0.07)***		0.06 (0.07)
Exercises regularly				1.34 (0.06)***		0.82 (0.06)***
Consumes vegetables				1.90 (0.09)***		1.53 (0.08)***
Consumes fish				0.64 (0.06)***		0.45 (0.05)***
Health Status						
Any chronic condition					−0.08 (0.05)	−0.19 (0.05)* **
Any ADL disability					−3.96 (0.08)***	−3.79 (0.08)***
Any IADL disability					−1.90 (0.06)***	−1.62 (0.06)***
Attrition (Ref: survivors)						
Loss to follow-up	−0.41 (0.08)***	−0.47 (0.08)***	−0.40 (0.08)***	−0.43 (0.08)***	−0.21 (0.08)***	−0.26 (0.08)***
Deceased	−2.49 (0.08)***	−2.45 (0.08)***	−2.47 (0.08)***	−2.32 (0.08)***	−1.73 (0.07)***	−1.62 (0.07)***
Goodness of fit						
AIC	248,552	243,980	248,485	247,188	244,433	239,279
BIC	248,681	244,151	248,631	247,317	244,588	239,493

*Abbreviations*: *AIC* Akaike Information Criterion, *BIC* Bayesian information criterion

Model 1: Adjusted for demographic characteristics; Model 2: Model 1 + Socioeconomic factors; Model 3: Model 1 + Family support; Model 4: Model 1 + Behaviroal factors; Model 5: Model 1 + Health status; Model 6: full model.

^a^centered at age 65; Standard errors within parentheses; * *p*-value < 0.05; ** *p*-value < 0.01; *** *p*-value < 0.001

**Fig. 2:**
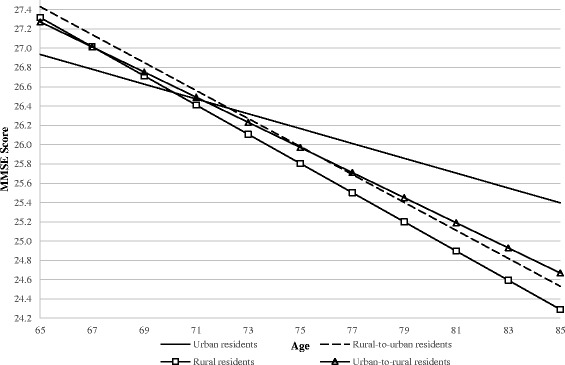
Predicted Linear Trajectories of MMSE scores among Chinese Older Adults by Residential Status

Model 2 included socioeconomic factors and shows changes in the trajectories of cognitive function related to residential status. At baseline, rural residents exhibited significantly better cognitive function at baseline compared with urban residents when accounting for socioeconomic factors. However, the results also showed that the initially higher MMSE scores among rural and rural-to-urban migrants decline at a significantly faster rate with age compared with long-term urban residents. Model 4 included behavioral factors and further showed that there were no significant baseline differences in cognitive function related to residential status. Additional analyses indicated that engaging in routine exercise was the key behavioral factor contributing to the attenuation of MMSE differences among residence groups at baseline. Model 6 included all covariates and indicated significant differences in the initial levels and/or changes in MMSE scores for rural and rural-to-urban residence relative to urban residence. To better illustrate these findings, Fig. 2 presents the results from these analyses. In sum, we found that urban residents had the lowest MMSE scores at baseline; however, we also found that older adults who resided in rural areas, migrated from rural-to-urban, or from urban-to-rural areas had significantly greater reductions in cognitive function with increasing age.

Several sensitivity analyses were also performed. First, the results were largely unchanged when including adults who were aged 90 and older at baseline—suggesting that selection bias at advanced ages did not play a significant role in the findings. Second, we also estimated three-level multilevel models to further include residential status as a random effect in the analyses (i.e., to account for possible nesting of respondent differences in each residential group). Third, we used baseline residential status as the predictor in the model and included a dummy variable that indicated whether the respondent moved or not during the course of the study. The results from these analyses were consistent with the findings reported here. Finally, we added a variable that indicated whether the current residential location is the same as their birthplace to account for potential urbanization in rural-to-urban resident group. The results were essentially similar to those we presented in this study.

## Discussion

The purpose of this study is to examine the association between early-life and adult residential status and changes in cognitive function over time. Using longitudinal data and a time-vary indicator of residential status for older adults in China, we found significant differences in the initial levels and rates of decline in cognitive function among older adults with different residential status. Furthermore, the relationships were not fully explained by older adults’ demographic background, socioeconomic resources, family support, health behaviors, and health status. The current study extends previous findings by further demonstrating how current residence and past migration can have a lasting association on trajectories of cognitive function in older adulthood [60, 63].

After adjusting for all covariates, we found that rural residents had better cognitive function at baseline but a faster rate of decline than urban residents, which partially supports our first hypothesis. The observed difference in the initial level of cognitive function may be related to selective survival; whereas, the faster rate of decline may be associated with the negative consequences of living in rural areas that have been documented in previous research [64]. We also found no difference in the initial levels nor rates of decline in cognitive function between urban-to-rural residents and urban residents. This finding is somewhat in contrast to our second hypothesis. However, based on the limited evidence of urban-to-rural residents in prior studies and the relatively small size of this group, more research is needed to explore whether moving from an urban residence to a rural one could have an impact on later-life cognitive function.

The findings from this study provide only partial support to the healthy migrant hypothesis [13]. Consistent with research in India [28], we found that rural-to-urban residents had better baseline cognitive function than urban residents in China. Although this finding does not fully support our second hypothesis, the explanation for this association may be attributable in part to the selection of healthy individuals who may be more likely and/or able to migrate [65]. Indeed, we found no difference in baseline MMSE scores between urban residents and rural-to-urban residents after taking into account the participants’ health status.

Contrary to the healthy migrant effect, however, we also found that rural-to-urban residents had more rapid declines in cognitive function than their urban counterparts. This finding partially supports our second hypothesis. Although adjustments for a variety of important confounders did not explain this association, it is possible that the cumulative toll of these risk factors at earlier ages may have precipitated an acceleration of cognitive decline at older ages. This argument is compatible with a large body of research on the life course which demonstrates that early-life and long-term exposure to disadvantage can have consequences across the adult life span [66–69]. In the context of China, previous research has shown that living in rural areas in early life is negatively associated with cognitive function [64]. In addition, it may be that migrating from rural to urban areas may limit an individual’s social network and available sources of social support from friends and family members. Studies have also suggested that rural-to-urban residents may be denied access to some of the social benefits available to their urban counterparts (e.g., retirement pension, health insurance, etc.), which may have important implications for access to health care and health maintenance [10].

An important area for future research will be to investigate the mechanisms underlying the findings from this study and to develop possible intervention strategies to reduce these risks. As expected, health status was the strongest correlate of cognitive function in our sample of older adult. Our analyses also suggested that socioeconomic and behavioral factors had among the greatest overall model fit for estimating the changes in cognitive function across age. Accordingly, we found that education, occupation, and economic independence were strongly associated with cognitive function among older adults in China. This finding is consistent with other studies showing that higher levels of SES were associated with higher overall levels of cognitive function [60, 63]. We also found that regular exercise, and healthy diet (consuming vegetables and fish) were significantly associated with higher cognitive performance, as shown elsewhere [38, 70] .However, our results suggest that health status, socioeconomic factors, and health behaviors appear to be operating largely independent of the migration variables. Therefore, we encourage additional quantitative and qualitative studies of the possible mechanisms at play.

This study has several limitations that should be acknowledged. First, the measure of residential status in the CLHLS is relatively crude. In particular, we were unable to identify the respondent’s age at migration, the reason(s) that they migrated, and multiple migrations—factors that may be important for understanding the association with cognitive health. Second, because of China’s urbanization in recent decades, we recognize that some urban areas may have been rural areas in the past. Therefore, it is possible that some of the findings related to change of residential status may be attributable to urbanization. Although we did a sensitivity analysis to account for potential urbanization, we are not able to fully differentiate rural-to-urban migrants from those whose residential status changed because of urbanization. It is possible that the underlying mechanisms for these two groups may be different. For example, those whose residential status changed because of urbanization might not experience separation from family or changes in their environment. However, they may also have similar experience in terms of raising living standard, change of lifestyle, and improved access to care. We encourage future studies to consider how these and other factors may contribute to changes in cognitive function related to residential status. Third, we acknowledge that the CLHLS includes the oldest-old adults in China and thus may include some degree of bias related to selective survival. However, this issue is largely unavoidable when studying cognitive decline at later ages.

Overall, this study provided a better knowledge and understanding on the risks related to decline of cognition. Results from this study also provided guidance for future research that addresses health disparities. Future research should not only compare rural versus urban residents, but also take consideration of the migrants, a large segment of the population in developing countries, the study results are particularly useful for those countries that are undergoing a significant social transition and urbanization. In addition, this study provided insights that help us target these identified risk factors in future research, and ultimately develop effective programs and interventions to improve cognitive function.

## Conclusions

In conclusion, the current study found a robust association between early-life and adult residential status and changes in cognitive function among older adults in China. These findings provide new evidence to a growing literature on the importance of social determinants in healthy aging. We encourage additional studies to corroborate the results from this study and further explore the factors that may be contributing to differential declines in cognitive health related to place of birth and adult living environments.

## Additional file

Additional file 1: Table S1. Parameter Estimates for Trajectories of Cognitive function Over Time. (DOCX 35 kb)

## Abbreviations

ADL: Activity of daily living
CLHLS: Chinese Longitudinal Healthy Longevity Survey
IADL: Instrumental activity of daily living
MCI: Mild cognitive impairment
MMSE: Mini Mental State Examination
SD: Standard deviation
SES: Socioeconomic status
