# Supplementary material for: The impact of residential status on cognitive decline among older adults in China: Results from a longitudinal study
Source: BMC Geriatr. 2017 May 15;17:107. doi: 10.1186/s12877-017-0501-9 (PMC5430605; doi:10.1186/s12877-017-0501-9)
Supplement: Additional file 1: Table S1. — Parameter Estimates for Trajectories of Cognitive function Over Time. (DOCX 35 kb) [file 12877_2017_501_MOESM1_ESM.docx]

**Additional file**

| **Additional file 1: Table S1**. Parameter Estimates for Trajectories of Cognitive function Over Time | | | | | | | |
| --- | --- | --- | --- | --- | --- | --- | --- |
|  | Model 1 | | Model 2 | | Model 3 | |  |
| **Fixed-Effects Parameters** |  |  |  |  |  |  |  |
| Intercept | 26.64^***^ | (26.57 to 26.70) | 29.46^***^ | (29.35 to 29.56) | 28.05^***^ | (27.90 to 28.19) |  |
| Age |  |  | -0.30^***^ | (-0.31 to -0.29) | 0.04^**^ | ( 0.02 to 0.07) |  |
| Age^2^ |  |  |  |  | -0.013^***^ | (-0.014 to -0.012) |  |
| **Random-Effects Variance Components** |  |  |  |  |  |  |  |
| Level 1: Within-person | 4.78^***^ | (4.74 to 4.82) | 4.69^***^ | (4.65 to 4.73) | 4.62^***^ | (4.58 to 4.66) |  |
| Level 2: Intercept | 1.93^***^ | (1.75 to 2.13) | 0.84^***^ | (0.70 to 1.01) | 0.81^***^ | (0.68 to 0.98) |  |
| Level 2: Slope | 0.38^***^ | (0.37 to 0.39) | 0.28^***^ | (0.27 to 0.29) | 0.28^***^ | (0.27 to 0.29) |  |
| **Goodness-of-Fit** |  |  |  |  |  |  |  |
| BIC value | 253773 | | 249615 | | 249851 | |  |
| Abbreviations: BIC, Bayesian information criterion.  Note: Estimated coefficients (95% confidence intervals) are reported. Age and Age^2^ indicate linear and quadratic slope parameters, respectively.  ^*^ *p* < 0.05, ^**^ *p* < 0.01, ^***^ *p* < 0.001 | | | | | | | |
